# Psychometric Properties of the Verbal Affective Memory Test-26 and Evaluation of Affective Biases in Major Depressive Disorder

**DOI:** 10.3389/fpsyg.2020.00961

**Published:** 2020-06-05

**Authors:** Liv V. Hjordt, Brice Ozenne, Sophia Armand, Vibeke H. Dam, Christian G. Jensen, Kristin Köhler-Forsberg, Gitte M. Knudsen, Dea S. Stenbæk

**Affiliations:** ^1^Neurobiology Research Unit and Center for Integrated Molecular Brain Imaging, Rigshospitalet, Copenhagen, Denmark; ^2^Department of Public Health, Section of Biostatistics, University of Copenhagen, Copenhagen, Denmark; ^3^Faculty of Health and Medical Sciences, University of Copenhagen, Copenhagen, Denmark; ^4^Centre for Mental Health Promotion, Department of Psychology, University of Copenhagen, Copenhagen, Denmark; ^5^Psychiatric Center Copenhagen, Copenhagen University Hospital, Rigshospitalet, Copenhagen, Denmark

**Keywords:** VAMT-26, VAMT-24, immediate recall, short-term memory, long-term memory, major depression disorder, affective memory biases

## Abstract

We developed the Verbal Affective Memory Test-26 (VAMT-26), a computerized test to assess verbal memory, as an improvement of the Verbal Affective Memory Test-24 (VAMT-24). Here, we psychometrically evaluate the VAMT-26 in 182 healthy controls, examine 1-month test–retest stability in 48 healthy controls, and examine whether 87 antidepressant-free patients diagnosed with Major Depressive Disorder (MDD) tested with VAMT-26 differed in affective memory biases from 335 healthy controls tested with VAMT24/26. We also examine whether affective memory biases are associated with depressive symptoms across the patients and healthy controls. VAMT-26 showed good psychometric properties. Age, sex, and IQ, but not education, influenced VAMT-26 scores. VAMT-26 scores converged satisfactorily with scores on a test associated with non-affective verbal memory. Test–retest analyses showed a learning effect and a *r* ≥ 0.0.8, corresponding to a typical variation of 10% in recalled words from first to second test. Patients tended to remember more negative words relative to positive words compared to healthy controls at borderline significance (*p* = 0.06), and affective memory biases were negatively associated with depressive symptoms across the two groups at borderline significance (*p* = 0.07), however, the effect sizes were small. Future studies are needed to address whether VAMT-26 can be used to distinguish between depression subtypes in patients with MDD. As a verbal memory test, VAMT-26 is a well validated neuropsychological test and we recommend it to be used in Danish and international studies on affective memory.

## Introduction

While verbal memory is a broad concept referring to memory for verbally presented information, verbal affective memory refers to memory for verbally presented information with an emotional content. Examination of non-affective and affective verbal memory typically involves the presentation of word lists or stories, which are subsequently recalled or recognized within a set time interval. Verbal memory is one of the most examined cognitive domains and considered fundamental to intelligence testing and to disease assessment and diagnosing (e.g., Alzheimer’s disease), as well as in the study of affective biases in cognition following psychological or pharmacological interventions (Lezak et al., 2004). However, currently, our knowledge about the interaction between affectivity and verbal memory is more limited (Groeger, 1997; Terry, 2003; Joormann and Stanton, 2016). The notion of a mood-congruent memory bias was first suggested by Bower (1981), who theorized that individuals show superior memory for material that is consistent with the individual’s mood state compared to material that is mood incongruent. Such mood-congruent memory bias may contribute to difficulties using adaptive emotion regulation strategies, and may also affect individuals’ perception of a certain situation and change their appraisals [as discussed in Joormann and Stanton (2016)]. Empirical evidence of depression-related memory biases, where clinically depressed individuals show a preferential recall of negative compared to positive information (Matt et al., 1992; Mathews and MacLeod, 2005), is especially supported by studies examining implicit (Gaddy and Ingram, 2014) and autobiographical (Kohler et al., 2015) mood-congruent memory. However, empirical evidence of a mood-congruent memory bias in explicit non-self-referential memory is more discrepant, as some studies provide support of a negative bias in patients with Major Depression Disorder (MDD) (Watkins et al., 1992; Bradley et al., 1995; Neshat-Doost et al., 1998), while other studies provide support of a positive bias (Danion et al., 1995; Calev, 1996; Zupan et al., 2017). Further, studying the association between verbal memory biases and depression may contribute to existing knowledge and enhance our understanding of affective cognition in affective disorders, as well as in general.

Unfortunately, most available verbal memory tests suffer from several methodological shortcomings, which especially holds true for tests of verbal affective memory (Lezak and Lezak, 2004). For example, the Affective Auditory Verbal Learning Test (AAVLT) (Snyder and Harrison, 1997) administered separate positive and negative word lists to separate individuals, despite it being more efficient to examine affective memory by administering positive, negative, and neutral information to the same individuals (as recommended by e.g., Elliott et al., 2011). The Emotional Verbal Learning Test (EVLT) (Strauss and Allen, 2013) consists of only 16 words, which increases the risk of ceiling effects (i.e., that all or nearly all words are recalled) and uses an unequal distribution of positive and negative words (i.e., 4 positive and 12 negative words), lowering the sensitivity for assessing memory for positive information. The Cognitive-Affective Verbal Learning Test (C-AVLT) (Considine et al., 2017) was developed to overcome several of the shortcomings of the AAVLT and the EVLT, for example. However, as for the EVLT, the C-AVLT consists of only 16 words (i.e., 4 positive words, 4 negative words, 4 neutral-abstract words, and 4 neutral-concrete words, increasing the risk of ceilings effects. In general, most existing verbal memory tests include a mix of common, highly unusual or taboo words, though the latter are known to affect memory performance differentially (Lezak and Lezak, 2004).

Taken together, new tests of recall of commonly encountered non-affective and affective words are needed (Elliott et al., 2011; Bayer and Schacht, 2014). To address some of the methodological shortcomings of existing verbal affective memory tests, we recently developed the Verbal Affective Memory Test-24 (VAMT-24), a computerized test to assess affective verbal memory (Jensen et al., 2015). Subsequently, we developed the Verbal Affective Memory Test-26 (VAMT-26) as a logical and theoretical improvement of VAMT-24, specially addressing the potential effects of word class on recall and a suboptimal proportion of affective words in VAMT-24. Compared to VAMT-24, VAMT-26 includes two more words to increase test difficulty and only nouns to control for potential memory enhancing effects of word class. Finally, VAMT-26 comprises a larger proportion of affective words to increase sensitivity in detecting affective biases (10 positive, 10 negative, and 6 neutral words).

In Part 1 of the study, we psychometrically evaluate VAMT in an extended 26-word version. In Part 2, we examine the impact of the adjustments from VAMT-24 to VAMT-26 on recall outcomes and propose a conversion algorithm to render VAMT scores comparable across different versions of VAMT. In Part 3, we examine biases in verbal affective memory in patients diagnosed with MDD and compare the biases to that of healthy controls.

## Part 1. Psychometric Properties of VAMT-26

In this part of the study, we investigate the psychometric properties of VAMT-26 in a large sample of healthy controls and assess the test–retest stability after approximately 1 month.

Based on our previous VAMT-24 validation (Jensen et al., 2015), we hypothesize that (1) the distribution of VAMT-26 outcomes can be approximated by a normal distribution, (2) learning effects occur over five consecutive immediate recall (IMR) trials, (3) mean recall rates of words presented in the beginning (primacy section) and at the end (recency section) of the test will be higher compared to words presented in the middle section, and (4) VAMT-26 outcomes are positively associated with an established neuropsychological instrument assessing verbal memory.

### Materials and Methods

#### Procedures and Participants

VAMT-26 data were acquired as part of other ongoing studies and stored in the Center for Integrated Molecular Brain Imaging (CIMBI) database. For descriptions of the CIMBI database, please see Knudsen et al. (2016). We extracted data from the CIMBI database, including healthy individuals between 18 and 65 years of age with VAMT-26 data from a first VAMT-26 measurement, and who did not undergo any experimental interventions. All individuals completed VAMT-26 in accordance with standardized VAMT-26 test administration procedures (Jensen et al., 2015). A total of 182 healthy individuals were eligible for the current study.

Across studies, exclusion criteria were a family history of neurological or primary psychiatric disorders (DSM IV Axis I or WHO ICD-10 diagnostic classifications), severe neurological or somatic illness, use of medication which could influence cognitive performance, learning disabilities, sight or hearing impairment, pregnancy, and substance and drug abuse (lifetime use of cannabis >50 times or lifetime use of any other drug >10 times). None of the healthy individuals presented with clinical levels of depression according to established Danish criteria for clinical cut off scores on the Major Depression Inventory > 21 (Olsen et al., 2004). All individuals were recruited by advertisement for different research protocols approved by the Ethics Committee of Copenhagen and Frederiksberg, Denmark (protocol numbers: H-15013578 (*n* = 97), H-3-2013-100 (*n* = 39), H-2-2014-070 (*n* = 12), H-15001910 (*n* = 8), H-16026898 (*n* = 17), H-15017713 (*n* = 8) and H-1-2014-002 (*n* = 1). After receiving verbal and written information about the respective studies, written informed consent was obtained prior to participation for all individuals. The included data was collected in the period from 2013 to 2018.

#### Verbal Affective Memory Task-26 (VAMT-26)

**FIGURE 1:**
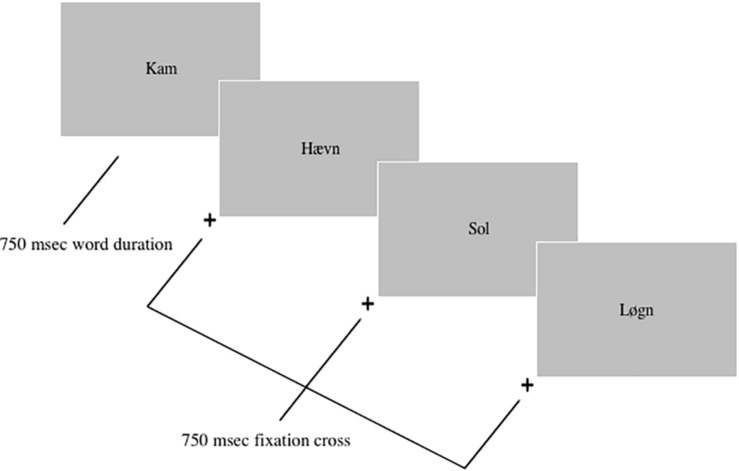
Illustration of the Verbal Affective Memory Test-26. Each word trial displayed a fixation cross (750 ms) and a word (750 ms) in black (font = Times, size = 40) on a gray background. The Danish [English] words presented in the figure are: Kam [comb], Haevn [revenge], Sol [sun], Løgn [lie].

VAMT-26 consists of 26 nouns: 10 positive, 10 negative, and 6 neutral. The basic task design, test administration and instructions of VAMT-26 are identical with that of VAMT-24. Participants are initially informed that a series of words (list A-26) will be presented several times on the computer screen and are asked to remember as many words as possible. The procedure is repeated five times (yielding the IMR score = IMR1 + IMR2 + IMR3 + IMR4 + IMR5), yet the participants are blinded to the number of recall repetition trials. The recalled words and mistakes (i.e., words that were not presented and thus, are incorrectly “recalled”) are noted on a preformed VAMT-26 scoring sheet. Following the IMR trials, the interference list (I-26) is displayed, after which, participants are asked to recall list A-26 without seeing it, yielding short-term memory (STM) scores. After a wait period of 30 min in which other cognitive tests are administered, participants are asked to do a surprise recall of list A-26, providing long-term memory (LTM) scores. The duration of a VAMT-26 test without the wait period is approximately 25 min. The valence of all words included in list A-26 and I-26 have previously been validated (Jensen et al., 2015). Based on the extraction of a count of each word’s occurrence in a linguistic research database^1^, we ensured that the overall frequency of use was not different between A-26 and I-26 and between valences in A-26. The words are displayed in a fixed order in regards to valence (1 = Positive, 2 = Negative, 3 = Neutral): 3-2-1-2-2-1-1-2-3-3-1-1-2-2-3-2-2-3-2-2-1-1-2-2-1-3. Words with similar first letters are separated by at least four other words in A-26 and I-26. Figure 1 shows an illustration of word presentation in VAMT-26. Each word is displayed for 750 ms on a computer screen, at a distance of approximately 60 cm, followed by an Interstimulus Interval (ISI) of 750 ms, displaying a fixation cross. VAMT-26 is programmed in Eprime 2.0 Professional (Psychology Software Tools, United States).

##### VAMT-26 outcomes

We defined 9 VAMT-26 outcomes; the total number of words recalled across IMR trials 1–5 (i.e., IMR1 + IMR2 + IMR3 + IMR4 + IMR5), and within STM and LTM (e.g., LTM Total), respectively, as well as the number of positive or negative words recalled across IMR trials 1–5 and within the STM trial and the LTM trial (e.g., LTM Positive), respectively.

#### Neuropsychological Tests

To measure Intelligence Quotient (IQ), we used Reynold’s Intellectual Screening Test (RIST) (Reynolds and Kamphaus, 2003). To examine convergent validity of VAMT-26, we used non-affective neuropsychological tests known to be related to verbal memory; Letter-Number Sequencing (LNS) from the Wechsler Adult Intelligence Scale-III (WAIS-III) (Wechsler, 1997). From these tests, we extracted the following main outcomes: *RIST index:* expressed as an age-adjusted standard IQ score; *LNS*: total number of correctly repeated series (scores range from 0–21). A more detailed description of these neuropsychological tests can be found in Jensen et al. (2015).

#### Data Analyses

##### Descriptive statistics

We visually inspected VAMT-26 histograms and P–P plots of the data with tests of normality (Shapiro–Wilk). For outcomes with non-normal distributions, the median and interquartile range are reported instead of the mean and standard deviation (SD).

##### Psychometric properties

Learning and recall effects: To evaluate learning effects, we examined changes in mean word recall between each IMR list presentation (i.e., comparing IMR1 to IMR2, IMR2 to IMR3, IMR3 to IMR4, IMR4 to IMR5) with seven Wilcoxon signed-rank tests. In addition, we examined whether presentation of the I-26 list significantly decreased STM Total compared to IMR5, and whether the 30 min interval between STM and LTM trials significantly decreased LTM Total compared to IMR5 and STM Total. **Primacy and recency effects:** We divided the A-26 list into three sections: primacy section = words number 1–3; middle section = words number 4–23; recency section = words number 24–26. To test primacy and recency effects, we examined differences in (Mean) percentage of words recalled across the five IMR trials between primacy section and middle section and between middle section and recency section, with two paired *t*-tests. **Internal consistency:** We examined internal consistency with nine Pearson product-moment correlation coefficients between each valence for IMR, STM, and LTM performances. **Test inherent affective biases:** We tested whether VAMT-26 exhibits test-inherent affective biases by comparing recall for positive and negative words within IMR, STM, and LTM respectively using three Wilcoxon signed-rank tests. **Ceiling effects:** We evaluated ceiling effects of VAMT-26 outcomes as a recall mean less than 1.5 SD from a maximum observed score (e.g., the maximum observed score for IMR Positive), yielding a standardized distance score.

##### Established covariates for verbal recall

We examined whether age, sex, IQ, and educational level are associated with each of the nine VAMT-26 outcomes in nine multiple regression models.

##### Convergent validity

To evaluate convergent validity, we examined the relations between VAMT-26 Total outcomes and LNS with nine Pearson product-moment correlation coefficients.

##### Test–retest analyses

To examine test–retest stability, a sub-group of the full sample (*n* = 48) were administered VAMT-26 on two occasions, with the two test sessions separated by approximately 1 month (Mean = 27.7 days, range: 21–43). Retest data was not included in the analyses evaluating the psychometric properties of VAMT-26. Stability was assessed for the IMR Total, STM Total, and LTM Total score using the Bland-Altman method (Bland and Altman, 1986; see Giavarina, 2015) for a more recent introduction. This method considers two components to assess stability: unbiasedness (referred to as learning effect hereafter) and precision (small variance or degree of scatter). We expressed the learning effect as the mean difference in total number of words recalled between the first and second test. The precision was defined as the half width of the 90% limits of agreement (LOA) interval, i.e., ignoring a possible learning effect, the interval [−precision; +precision] contains the difference in words between the test and the retest of 90% of the observations. We chose 90% instead of the traditional 95% to better reflect the typical sampling error. To be consistent with the existing literature, we also report Pearson’s correlation coefficient as another measure of precision.

##### Correction for multiple comparisons

Unless otherwise stated, *p*-values were adjusted with the Bonferroni–Holm multiple comparison procedure (Holm, 1979), with the number of statistical tests carried out. An alpha level of 0.05 was adopted throughout all analyses. Statistical analyses were conducted using Statistical Package for the Social Sciences version 25.0 (SPSS).

### Results

#### Descriptive Statistics

Descriptive information about the 182 healthy individuals included in Part 1 of the study is presented in Table 1. Descriptive information on VAMT-26 outcomes at the first test is displayed in Table 2. The mean IQ score was in the high end of the normal range. The normal distribution provided a reasonable approximation to the distribution of IMR Total, IMR Positive, and IMR Negative (Shapiro Wilks *ps* > 0.09). Other VAMT-26 outcome distributions were left-skewed (Shapiro Wilks *ps* < 0.05). Missing values were: Education: *n* = 3, LNS *n* = 2, and BMI: *n* = 44. BMI were not acquired in all studies from which the data from the CIMBI database originate, explaining the high number of missing values.

**TABLE 1:** Descriptive information for the healthy sample in Part 1.

	All (*N* = 182)		
	*n*	*%*	
Sex, females	*79*	43.4	
	***Mean***	***SD***	***Range***
Age, years	29.4	7.8	18.8–54.5
BMI	23.7	3.2	17.5–35.3
IQ score	109.9	7.1	89–129
***Educational level (1–5)***	***n***	***%***	
Level 1	15	8.4	
Level 2	7	3.9	
Level 3	13	7.3	
Level 4	32	17.9	
Level 5	112	62.6	
***Cognitive data***	***Mean***	***SD***	***Range***
LNS	13.0	3.0	5–20

*Descriptive data for the Verbal Affective Memory Test-26 validation sample. Body mass index is reported in weight in kg/(height in squared meters), Intelligence Quotient score is assessed with the Reynolds Intellectual Screening Test, education score is measured with the Online Stimulant and Family History Assessment Module. SD, standard deviation; BMI, body mass index; IQ, intelligence quotient; LNS, letter-number sequencing.*

**TABLE 2:** Descriptive information on VAMT-26 outcomes.

All (*N* = 182)	*Mean*	*SD*	*Median*	*IQR*	*Range*	*Individuals with maximum scores (%)*	*Skewness*	*Standardized distance*
IMR Total	80.8	16.8	81.0	21	33–118	0	−0.24	−2.2
STM Total	17.3	4.8	17.5	7	4–26	2.8	−0.32	−1.8
LTM Total	17.8	4.6	19	7	6–26	2.8	−0.29	−1.8
IMR Positive	30.4	7.3	30	9	13–46	0	−0.10	−2.2
IMR Negative	30.1	7.1	30	9	10–45	0	−0.18	−2.1
STM Positive	6.3	2.1	6	3	2–10	7.1	−0.09	−1.7
STM Negative	6.4	2.2	6	3	1–10	7.3	−0.22	−1.7
LTM Positive	6.6	2.1	7	3	1–10	9.3	−0.31	−1.6
LTM Negative	6.4	2.1	7	3	2–10	8.2	−0.14	−1.7
**Women (*n* = 79)**	***Mean***	***SD***	***Median***	***IQR***	***Range***	***Individuals with maximum scores (%)***	***Skewness***	
IMR Total	85.5	15.3	87	*21*	41–118	0	−0.4	
STM Total	18.4	4.4	19	*6*	7–26	3.8	−0.5	
LTM Total	19.4	4.1	20	*6*	9–26	5.1	−0.5	
IMR Positive	31.8	6.8	32	8	13–45	0	−0.4	
IMR Negative	31.7	6.4	32	9	15–45	0	−0.3	
STM Positive	6.9	2.0	7	3	2–10	11.4	−0.1	
STM Negative	6.7	2.0	7	3	2–10	6.3	−0.3	
LTM Positive	7.4	1.9	8	3	2–10	13.9	−0.5	
LTM Negative	6.9	2.0	7	2	2–10	10.1	−0.4	
**Men (*n* = 103)**	***Mean***	***SD***	***Median***	***IQR***	***Range***	***Individuals with maximum scores (%)***	***Skewness***	
IMR Total	77.3	17.0	77	21	33–115	0	−0.1	
STM Total	16.5	4.9	17	7	4–26	1.9	−0.2	
LTM Total	16.7	4.6	17	7	6–26	1.0	−0.1	
IMR Positive	28.7	7.4	28	9	13–46	0	0.1	
IMR Negative	28.9	7.4	29	8	10–44	0	0.004	
STM Positive	5.9	2.1	6	4	2–10	3.9	−0.1	
STM Negative	6.1	2.3	6	4	1–10	7.8	−0.1	
LTM Positive	6.1	2.1	6	3	1–10	5.8	−0.1	
LTM Negative	6.0	2.2	6	4	2–10	6.8	0.1	

*SD, standard deviation, median; IQR, interquartile range, minimum and maximum scores, ceiling effects in percent and skewness scores for the primary outcomes of the Verbal Affective Memory Test-26. IMR, immediate recall; STM, short-term memory; LTM, long-term memory.*

#### Psychometric Properties

**FIGURE 2:**
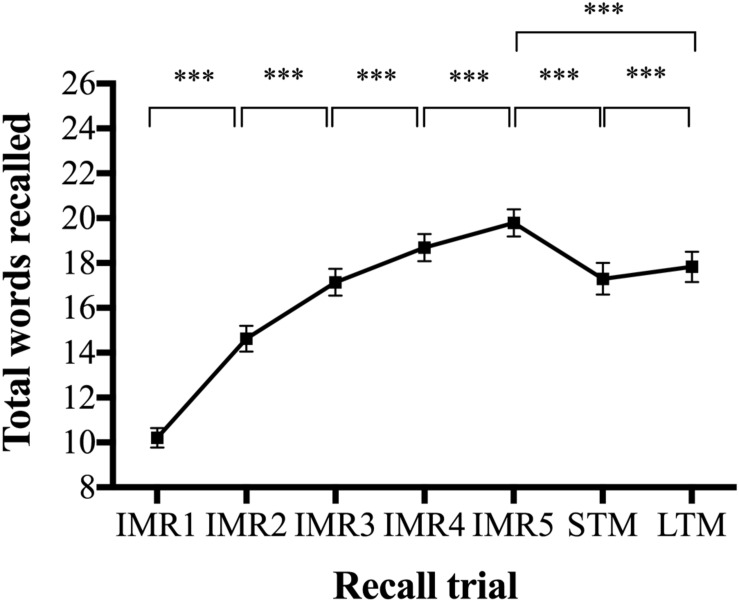
Recall curves for the Verbal Affective Memory Test-26 (VAMT-26). ***p < 0.0001. Recall means and confidence intervals (CI) for each of the seven trials in VAMT-26. Parametric tests were used to calculate the CI displayed in the figure. IMR1–5, immediate recall trials 1–5; STM, short-term memory; LTM, long-term memory. *P*-values in analyses on learning effects (i.e., change in total recall of words between each IMR list presentation) were obtained using Wilcoxon sign-rank tests and adjusted for four comparisons using the Bonferroni–Holm adjustment procedure (Holm, 1979). *P*-values in analyses on differences in recall between IMR5 vs. STM, IMR5 vs. LTM trials, STM vs. LTM were obtained using three Wilcoxon sign-rank tests and adjusted for three comparisons using the Bonferroni–Holm adjustment procedure (Holm, 1979).

**FIGURE 3:**
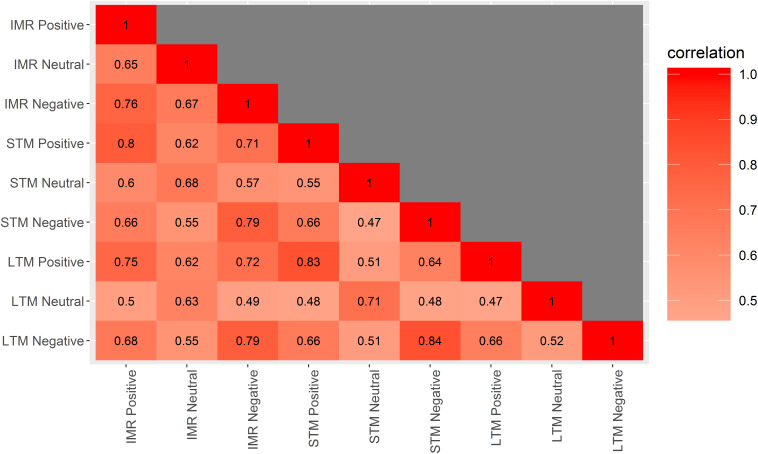
Correlations between each valence for IMR, STM, and LTM VAMT-26 performances. Correlation matrix plot showing Pearson product-moment correlation coefficients between all valences (i.e., positive, negative, and neutral) within IMR (i.e., words recalled across IMR trials 1–5, i.e., IMR1 + IMR2 + IMR3 + IMR4 + IMR5), STM, and LTM. IMR, immediate recall; STM, short-term memory; LTM, long-term memory.

Figure 2 shows recall curves for VAMT-26, and Figure 3 shows Pearson product-moment correlation coefficients between IMR, STM, and LTM valences. Descriptive information for IMR 1–5 recall trials and for primacy and recency effects are listed in Supplementary Table S1. **Learning effects:** Absolute recall of words increased significantly between each IMR list presentation (median difference range: 1–5, *Z range* = −5.8 – −11.3, *ps* < 0.001) (Figure 2 and Supplementary Table S1). Recall of words within the STM trial was significantly lower compared to recall of words within the IMR5 trial (median difference = −2.5, *Z* = −9.5, *p* < 0.001). The 30 min interval significantly decreased recall of words within the LTM trial compared to the IMR5 trial (median difference = −1.0, *Z* = −8.6, *p* < 0.001) but increased recall of words within the LTM trial compared to the STM trial (median difference = 1.5, *Z* = −3.8, *p* < 0.001). In *post hoc* analyses, we examined differences in STM and LTM recall within positive and negative words using Wilcoxon signed-rank analyses. We found a significant increase in recall for positive (median difference = 1.0, *Z* = −3.6, *p* < 0.001, unadjusted), but not for negative words (difference = 1.0, *Z* = −0.88, *p* = 0.38, unadjusted). **Primacy and recency effects:** Recall of the first three presented words (primacy section) was significantly higher than recall of the middle section of twenty words (median% difference = 28.2, *t* = 21.7, *p* < 0.001). The recall of the last three presented words (recency section) was also significantly higher than recall of the middle section (median% difference = 8.2, *t* = −6.2, *p* < 0.001).

##### Internal consistency

IMR, STM, and LTM scores were significantly associated within all valences: positive words (*r* range = 0.75–0.83, 95% CI range = 0.68–0.88), negative words (*r* range = 0.79–0.84, 95% CI range = 0.72–0.88) and neutral words (*r* range = 0.63–0.71, 95% CI range = 0.52–0.79) (Figure 3).

##### Test inherent affective biases

Recall of positive and negative words was not significantly different within IMR (*Z* = −0.2, *p* = 0.87), STM (*Z* = −0.9, *p* = 0.75) and LTM (*Z* = −1.77, *p* = 0.23). **Ceiling effects:** All standardized distance scores for VAMT-26 outcomes were above −1.6 SD from maximum score. In addition, 7% of the participants recalled all positive and negative words within STM, 9% recalled all positive words within LTM and 8% recalled all negative words within LTM (Table 2).

#### Established Covariates for Verbal Recall

Associations between VAMT-26 outcomes and established covariates are listed in Supplementary Table S2. Young adults showed superior recall across all VAMT-26 outcomes [beta coefficients (β) range: −0.64 – −0.05, *ps* ≤ 0.01). Women showed superior LTM Total (β = −1.86, *p* = 0.03) compared to men, which was driven by LTM Positive (β = −0.96, *p* = 0.01). Sex was not associated with performance on other VAMT-26 outcomes (*p*s ≥ 0.15). Higher IQ was associated with better recall of all VAMT-26 outcomes (β range: 0.05–0.64, *p*s ≤ 0.04), except for STM Negative, which was of borderline significance (*p* = 0.08). Educational level was not associated with any VAMT-26 outcomes (*ps* ≤ 0.34).

#### Convergent Validity

Total numbers of words recalled within IMR, STM, and LTM, respectively, were positively associated with scores on LNS (*r* range = 0.21–0.23, mean *r* = 0.22).

#### Test–Retest Analyses

Bland and Altman plots comparing the scores between the first and second test for IMR Total, STM Total, and LTM Total are presented in Supplementary Figure S3.

Results on learning effects showed that the mean difference (the bias) for total number of words recalled between the first and second test was: IMR Total = 16.5 (95% CI = 13.9; 19.1), STM Total = 3.9 (95% CI = 3.1; 4.6), and LTM Total = 3.1 (95% CI = 2.4; 3.8). The results of the bias indicated a significant increase in recall at the second test session and supported a learning effect. The half width of the 90% LOA interval was of 11.6 words for IMR Total, 3.2 words for STM Total, and 3.1 words for LTM Total. With respect to the achievable IMR Total score (range from 0–130) and the achievable STM Total and LTM Total scores (range from 0–26), this corresponds to a difference in remembered words between first test and second test of 8.9% for IMR Total, 12.2% for STM Total and 11.9% for LTM Total. The corresponding Pearson product-moment correlation coefficients were all large: *r* = 0.79 for IMR Total, *r* = 0.81 for STM Total, and *r* = 0.80 for LTM Total.

## Part 2. Comparison of VAMT-24 and VAMT-26

In this part of the study, we examine the impact of change in VAMT versions on VAMT recall outcomes and propose an adjustment procedure to make recall performance independent of VAMT version.

### Materials and Methods

#### Procedures and Participants

To evaluate the impact of change in VAMT versions, we used data from the same *n* = 182 healthy individuals from Part 1 of the study. Of these individuals, three were excluded because of missing education scores. Thus, the final sample of healthy individuals with VAMT-26 data in Part 2 of the study was *n* = 179. In addition, we extracted data from the CIMBI database including healthy individuals between 18 and 65 years of age with baseline VAMT-24 data and who did not undergo any experimental interventions. This initial data extraction included VAMT-24 data from *n* = 166 healthy individuals. Of these individuals, three were excluded because of missing LTM scores, three because of missing IQ scores, and four because of missing education scores. Thus, the final sample of healthy individuals with VAMT-24 data was *n* = 156, of these *n* = 133 are reported in the VAMT-24 validation study (Jensen et al., 2015). One individual completed both a VAMT-24 test and a VAMT-26 test but was included in the analyses as there was a period of 4.3 years between completion of the two VAMT tests.

Exclusion criteria for individuals with a VAMT-24 test were similar as for individuals with a VAMT-26 test and are described in Part 1 of the study. All individuals with a VAMT-24 test were recruited by advertisement for different research protocols approved by the Ethics Committee of Copenhagen and Frederiksberg, Denmark [H-protocol, numbers: KF-01-2006-20 (*n* = 22), KF-01280377 (*n* = 3), H-1-2010-085 (*n* = 34), H-1-2010-091 (*n* = 13), H-3-2012-083 (*n* = 1), H-2-2010-108 (*n* = 64), H-4-2012-105 (*n* = 29)]. After a complete description of the respective studies, written informed consent was obtained prior to participation for all participants. The included data was collected from 2011 to 2013.

#### Data Analyses

##### Descriptive statistics

Differences in demographics between individuals with VAMT-24 and VAMT-26 data were evaluated with Wilcoxon signed-rank tests, while Fisher’s exact tests were used to examine differences in categorical data.

##### Defining VAMT recall rates

In addition to the nine VAMT recall outcomes described in Part 1, three additional outcomes were defined: number of neutral words recalled across the IMR trials 1–5 and within the STM trial and the LTM trial, respectively. Since VAMT-24 and VAMT-26 word lists consist of different numbers of words, we normalized the recall rates for VAMT-24 and VAMT-26 by dividing them by the maximum achievable score in the list, e.g., each individual’s VAMT-26 IMR Positive score is divided by 50 (the maximum score), whereas each individual’s VAMT-24 IMR Positive score is divided by 40 (the maximum score). This corresponds to the percentage of recalled words and is termed *recall-%* hereafter.

##### Impact of the change in VAMT-versions

For each recall outcome, a linear univariate Gaussian regression model was used to model its mean and its variance. The mean was modeled as a function of VAMT version, age, sex, IQ, and educational level (as a continuous variable). Age, IQ, and educational level were centered using the median and their effects on the recall score were modeled using a polynomial of degree 3. The variance was modeled as a function of VAMT version. Univariate regression models were chosen over multivariate regression models to avoid making assumptions about the correlation between recall-% scores for VAMT tests. The effect of age, sex, IQ, and educational level were constrained to be the same across VAMT versions. Diagnostic tests were performed to assess deviations from the normality assumption of the residuals of the univariate regression models. To test whether the mean and variance for each VAMT outcome differed between VAMT versions, we first calculated the difference in modeled mean for VAMT-26 recall-% vs. the modeled mean for VAMT-24% recall-%, as well as the ratio between modeled variances for VAMT-26 and VAMT-24 recall-%. Next, we tested whether the difference in modeled mean significantly differed from 0 and whether the logarithm of the ratio between modeled variances significantly differed from 0.

To investigate whether matching the distribution of VAMT-24 and VAMT-26 recall-% on their mean and variance were sufficient, we compared VAMT-24 with VAMT-26 recall-% (adjusted for age, sex, IQ, and educational level) by visual inspection of their histograms and using Kolmogorov-Smirnov tests.

We used robust Wald tests to evaluate significance levels, which makes our analyses and estimated confidence intervals robust to deviations to the normality assumption (White, 1982). *P*-values and confidence intervals were adjusted by the max-test procedure for multiple comparison procedure [single step, Dmitrienko and D’Agostino (2013)]. For this procedure, the covariance between the robust Wald statistics was estimated by computing the covariance between the influence function of the difference in modeled mean and of the logarithm of the ratio between the modeled variances over the nine univariate regression models (Pipper et al., 2012).

An alpha level of 0.05 was adopted throughout all analyses. Statistical analyses were conducted using R (version 3.3.0) (R Core Team, 2016).

### Results

#### Descriptive Statistics

Descriptive information about the individuals included in Part 2 are listed in Table 3. Individuals tested with VAMT-24 were, on average, younger and had lower IQ compared to individuals tested with VAMT-26, but the two groups did not differ significantly in terms of BMI and educational scores (*ps* > 0.37).

**TABLE 3:** Descriptive information for the healthy samples in Part 2.

	All VAMT-24 (*N* = 156)			All VAMT-26 (*N* = 179)		
	*Mean*	*SD*	*Range*	*Mean*	*SD*	*Range*
Age	24.7	5.2	18.4–46.1	29.4	7.8	18.8–54.5
IQ score	107.8	7.6	88–126	109.8	7.1	89–129
*Educational level (1–5)*	N	%		N	%	
Level 1	20	12.8		15	8.4	
Level 2	5	3,2		7	3.9	
Level 3	6	3.9		13	7.3	
Level 4	33	21.2		32	17.9	
Level 5	92	59		112	62.6	

*Descriptive data for individuals with VAMT-24 and VAMT-26 data. Body mass index is reported in weight in kg/(height in squared meters), Intelligence Quotient score is assessed with the Reynolds Intellectual Screening Test, education score is measured with the Online Stimulant and Family History Assessment Module. SD, standard deviation; BMI, body mass index; IQ, intelligence quotient.*

#### Impact of the Change in VAMT-Versions

**FIGURE 4:**
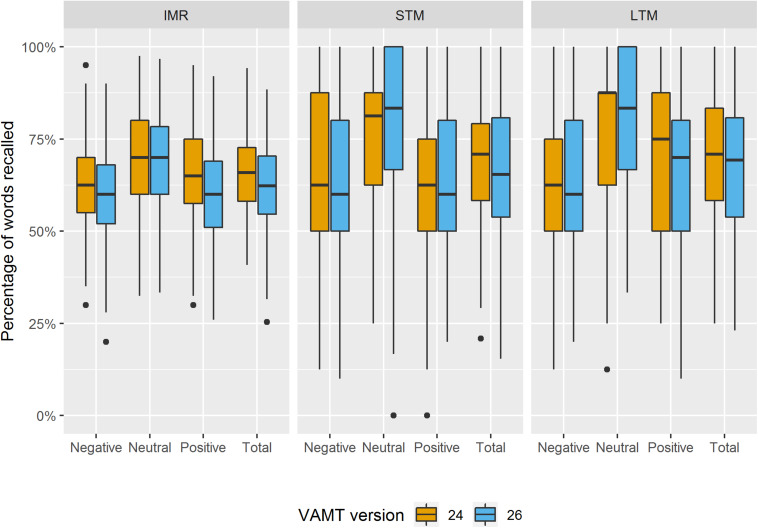
Boxplots of distribution of the percentage of VAMT-24 and VAMT-26 recall outcomes. The boxplots show the distribution of the percentage of words recalled in total and within each valence (i.e., Positive, Negative, and Neutral) across the IMR trials 1–5, the STM trial and the LTM trial for VAMT-24 and VAMT-26, respectively. Outliers are identified as scores that fall below first quartile – 1.5 × IQR or above third quartile + 1.5 × IQR. Outliers are plotted as individual points.

The distribution of the percentage of words recalled within each valence across the IMR trials 1–5, the STM and the LTM trial for VAMT-24 and VAMT-26, respectively, are displayed in Figure 4. The modeled means and variances of VAMT-24 and VAMT-26 recall-% outcomes (i.e., VAMT-24 and VAMT-26 recall-% scores adjusted for age, sex, IQ, and education) estimated by the univariate regression models are presented in Table 4. Q–Q plots of the residuals of the 12 univariate regression models are displayed in Supplementary Figure S1. Histograms of distribution of VAMT-24 and VAMT-26% recall rates, adjusted for age, sex, IQ, and educational level are shown in Supplementary Figure S2.

**TABLE 4:** Modeled means and variances for VAMT-24 and VAMT-26.

Outcomes	Mean%					Variance × 10^4^				
	*VAMT-24*	*VAMT-26*	*Difference*	*CI*	*P-value*	*VAMT-24*	*VAMT-26*	*Ratio*	*CI*	*P-value*
IMR Total	67.5	65.7	−1.9	−5.2; 1.4	0.50	107.9	125.9	1.2	0.8; 1.7	0.91
STM Total	73.1	72.2	−0.8	−5.8; 4.1	1.00	225.9	260.9	1.2	0.8; 1.7	0.95
LTM Total	75.5	75.4	−0.1	−5.1; 4.8	1.00	224.9	244.4	1.1	0.7; 1.6	1.00
IMR Positive	66.9	63.3	−3.6	−7.7; 0.5	0.11	156.7	170.7	1.1	0.7; 1.6	1.00
IMR Negative	64.2	64.2	0.04	−3.5; 3.6	1.00	125.9	156.9	1.2	0.8; 1.9	0.67
IMR Neutral	71.7	72.4	0.7	−3.1; 4.6	1.00	164.5	158.4	0.1	0.7; 1.4	1.00
STM Positive	68.2	69.6	1.4	−4.8; 7.6	1.00	380.1	346.0	0.9	0.6; 1.3	1.00
STM Negative	69.8	69.1	−0.6	−6.8; 5.5	1.00	378.3	411.3	0.05	0.8; 1.6	1.00
STM Neutral	80.9	82.0	1.1	−4.6; 6.9	1.00	314.2	402.0	1.3	0.9; 1.9	0.53
LTM Positive	74.7	76.5	1.7	−4.3; 7.7	0.97	370.0	357.7	1.0	0.7; 1.4	1.00
LTM Negative	70.4	68.9	−1.5	−7.7; 4.6	0.99	352.9	397.5	1.2	0.7; 1.7	0.99
LTM Neutral	80.9	84.9	4.0	−1.6; 9.6	0.28	348.1	291.4	0.8	0.6; 1.2	0.88

*Modeled means (respectively variances) of VAMT-24 and VAMT-26 recall outcomes, the difference in modeled means (resp. log of the modeled variance) between VAMT versions with its confidence intervals (CI), and the p-value for the null hypothesis of no difference. The modeled mean and variance were estimated using univariate linear regressions and adjusted for age, sex, Intelligence Quotient (IQ), and educational level. P-values and CI were adjusted using the max-test procedure [single step, Dmitrienko and D’Agostino (2013)].*

Deviations from the normality assumption of the residuals of the univariate regression models were observed for STM Neutral and LTM Neutral (Supplementary Figure S1). No significant differences between VAMT versions in modeled means or variances were observed (*ps* > 0.1) (Table 4).

The age, sex, IQ, and education adjusted distributions for VAMT-24 and VAMT-26 recall-% outcomes appeared similar. This was in line with the results of the Kolmogorov–Smirnov tests: *p*-values, unadjusted for multiple comparisons, were all above 0.10, except for IMR Positive (*p* = 0.03). See Supplementary Figure S2 for a graphical display of the discrepancy between the two distributions.

Our results suggest that data from VAMT-24 and VAMT-26 can be analyzed in a joint model by considering recall percentage (recall-%) scores instead of raw scores, i.e., by dividing the raw recall scores by the maximal achievable score within each VAMT recall rate and for each VAMT version.

## Part 3. Investigating Affective Memory Biases in Major Depression

In this part of the study, we examine biases in verbal affective memory in antidepressant-free patients diagnosed with MDD compared to healthy controls. We also evaluate whether VAMT Bias scores are associated with depressive symptoms across the two groups, i.e., including a sample of 422 individuals with a broad continuum of depressive symptoms, ranging from very low to very high.

### Hypotheses

In accordance with the hypothesis of a mood-congruent memory bias by Bower (1981), we explored whether (1) patients with MDD will display a negative memory bias (i.e., recall significantly more negative words relative to positive words) within IMR, STM, and LTM compared to healthy controls, and whether (2) VAMT Bias scores are negatively correlated with depressive scores, e.g., high negative VAMT Bias scores are associated with high depression severity.

### Materials and Methods

#### Procedures and Participants

We evaluated affective memory biases in MDD, using a sample of patients diagnosed with MDD as compared to the sample of healthy individuals from Part 2. Patients with MDD were recruited from general practitioners or from a central referral center within the mental health services in the capital region of Denmark. Exclusion criteria for patients with MDD included non-depressive psychiatric history or comorbidity, significant somatic illness, brain trauma, use of psychotropic medication, significant lifetime history of drug or alcohol abuse, and pregnancy or breastfeeding. Subsequent to initial screening, and to establish MDD diagnosis, MDD candidates were interviewed by a certified psychiatrist and evaluated by Mini-International Neuropsychiatric Interview (MINI). Symptom severity was assessed using the Hamilton Depression Rating Scale-17 (HDRS-17).

In a large multimodal neuroimaging study, that also includes neuropsychological testing, 100 medication-free patients with a moderate to severe major depressive episode according to the HDRS-17 (score ≥ 18) were included (Neuropharm). Neuropsychological testing was managed by trained testers and took place in standardized test rooms. Of the 100 patients with MDD included in the study, four patients dropped out prior to the neuropsychological examination, one patient spontaneously remitted before neuropsychological examination, one patient could not complete neuropsychological examination because of severe emotional distress, and two patients were pregnant at the time of the neuropsychological examination. Finally, five patients did not complete VAMT-26 because of non-fluency in Danish. Hence, a total of 87 individuals diagnosed with MDD and 335 healthy controls were included in Part 3. For all individuals, at the time of the neuropsychological testing, the subjective ratings of depressive symptoms were assessed with the Major Depression Inventory (MDI) assessing (Bech et al., 2001).

The Capital Region Ethics Committee approved the study (protocol: H-15017713) and was registered as a clinical trial at www.ClinicalsTrials.gov (protocol: NCT02869035). All participants signed informed consent prior to participation.

#### Data Analyses

##### Descriptive statistics and main outcomes

Differences in demographics between patients with MDD and healthy controls were evaluated with Wilcoxon signed-rank tests, while Fisher’s exact tests were used to examine differences in categorical data. We defined three VAMT Bias scores to test for affective memory biases: IMR Bias (IMR Positive words – IMR Negative words), STM Bias (e.g., STM Positive words – STM Negative words), and LTM Bias (e.g., LTM Positive words – LTM Negative words).

##### Affective memory biases

All VAMT Bias scores were converted into percentage scores according to the procedure described in Part 2. To examine our first hypotheses, we conducted three linear regression models to regress the effect of group (i.e., patients with MDD and healthy controls) on IMR Bias, STM Bias and LTM Bias, respectively. A Wald test was obtained from the three linear regression models to evaluate the significance level of an overall group effect on VAMT Bias scores across IMR, STM and LTM. Covariates for the linear regression model analyses included sex and age. Educational level was not included as covariate in the models as educational scores were not associated with any VAMT-26 outcomes. Nor was IQ score used as a covariate, as IQ tests were performed while patients were depressed, and hence potentially did not reflect their premorbid IQ scores. In order to test our second hypothesis, we applied three linear regression models to evaluate the association between IMR Bias, STM Bias and LTM Bias, respectively and MDI scores, correcting for age and sex. Again, a Wald test was obtained from the three linear regression models to evaluate the significance level of the overall association between VAMT Bias scores and MDI scores. We visually inspected deviations from the normality assumption of the residuals of the univariate regression models.

An alpha level of 0.05 was adopted throughout all analyses. Statistical analyses were conducted using R (version 3.3.0) (R Core Team, 2016) and SPSS (version 25.0).

### Results

#### Descriptive Statistics

The MDD and healthy control groups were not significantly different in age (*p* = 0.45); mean age in the MDD group: 27 years (SD = 7.8), mean age in the healthy control group: 27 years (SD = 7.1). The MDD and healthy control groups were significantly different in terms of sex distribution (*p* < 0.001): 71.3% females in the MDD group, 52.8% females in the healthy control group. The median of HDRS-17 score was 22.0 (IQR = 5, range 18–31 (assessed in patients with MDD only), indicating a moderate to severe major depressive episode at the time of VAMT-26 testing.

#### Affective Memory Biases

**FIGURE 5:**
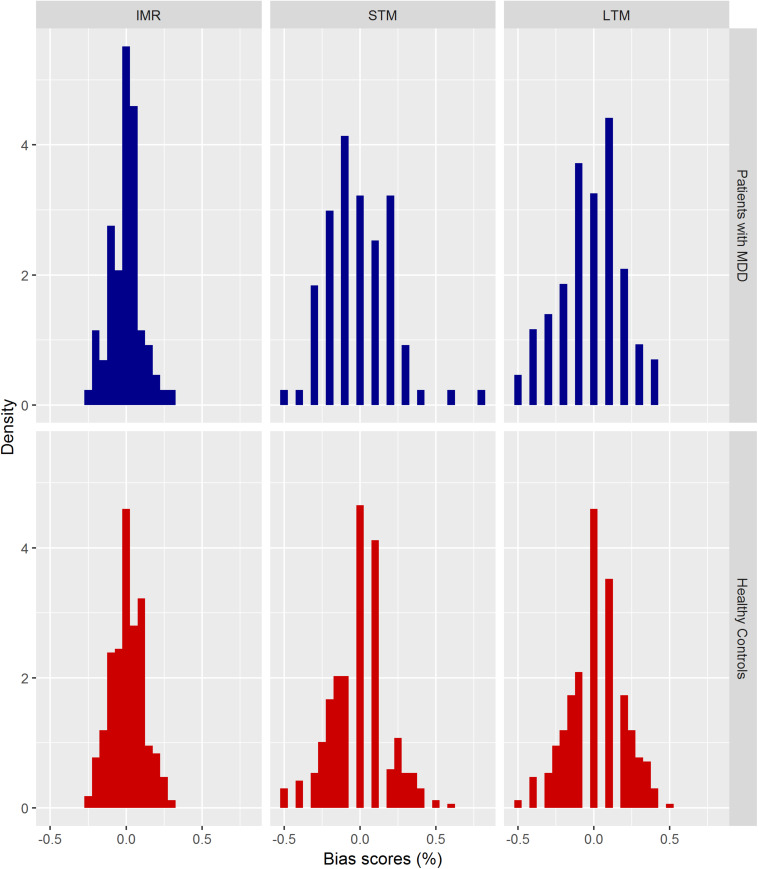
Distribution of VAMT Bias scores (in percentage) for patients with MDD and healthy controls. Histograms of the distribution of VAMT Bias scores adjusted for age and sex. The *y*-axis (density) indicates to the relative frequency normalized such that the area covered by the bars of the histogram equals 1.

No major deviations from the normality assumption of the residuals of the univariate regression models were observed for Bias scores within IMR, STM, and LTM. Mean VAMT Bias scores (in percentage) within IMR, STM, and LTM for patients with MDD and healthy controls are presented in Table 5. Histograms of the distribution of VAMT Bias scores adjusted for age and sex are shown in Figure 5.

**TABLE 5:** Descriptive information for VAMT biases.

Outcomes	Patients with MDD (*n* = 87)		Healthy controls (*n* = 335)	
	*Mean*	*SD*	*Mean*	*SD*
IMR Bias	−0.60	10.10	1.41	10.69
STM Bias	−0.92	21.60	−0.46	19.13
LTM Bias	−2.21	20.77	1.93	18.96

*Mean and standard deviation for VAMT biases (i.e., positive scores – negative score) in percentage within IMR, STM, and LTM. IMR, immediate recall; STM, short-term memory; LTM, long-term memory.*

We observed a main overall effect of group on VAMT Bias scores across IMR, STM, and LTM, at a statistical trend level (*p* = 0.06), where patients with MDD tended to recall more negative words than positive words compared to healthy controls who recalled more positive words compared to negative words. Although the main effect was only significant at trend level, we explored the statistical trend and looked at each of the three linear regression models separately. We found significant effects of group on IMR Bias (β = −0.03, *p* = 0.048 unadjusted, 95% CI [−0.05, −0.0003]), and LTM Bias scores (β = −0.05, *p* = 0.030 unadjusted, 95% CI [−0.01, −0.005]). The magnitude of bias in terms of words within IMR and LTM were 1.25 and 0.5 words, respectively. We did not observe an effect of group on STM Bias (β = −0.01, *p* = 0.69, unadjusted, 95% CI [−0.06, 0.04]).

We observed an overall main effect of IMR Bias, STM Bias, and LTM Bias on MDI scores, at a statistical trend level (*p* = 0.069), where higher negative VAMT Bias scores were associated with higher MDI scores. When limiting the analysis on VAMT Bias and the severity of depressive symptoms to the patients with MDD, the association was not significant (*p* = 0.75). Although, the main effect was only significant at trend level, we explored the statistical trend and looked at each of the three linear regression models separately. We found a significant negative association between MDI scores and IMR Bias (β = −11.4, *p* = 0.05 unadjusted, 95% CI [−23.1, 0.2]), and LTM Bias scores (β = −7.7, *p* = 0.016 unadjusted, 95% CI [−14.1, −1.4]). We did not observe an association between STM Bias and MDI scores (β = −3.0, *p* = 0.34, unadjusted, 95% CI [−9.2, 3.2]).

## Discussion

Verbal affective memory tests may yield a greater understanding of affective cognition and its relevance for psychological health. Motivated by methodological shortcomings in available tests of verbal affective memory, we developed an extended version of the previously validated VAMT-24 (Jensen et al., 2015), termed VAMT-26. The present study evaluated the psychometric properties of VAMT-26 within a large sample of healthy adults and examined affective memory biases in MDD. The VAMT-26 was hereby supported as a valid test of verbal affective memory with good psychometric properties, such as no ceiling effects. VAMT-26-scores also converged satisfactorily with scores on a neuropsychological test associated with non-affective verbal memory (i.e., LNS). Test–retest precision was satisfactory, while a significant increase in VAMT-26 Total scores at the second test session was observed, supporting a learning effect. Patients with MDD tended to remember more negative words relative to positive words compared to healthy controls at borderline significance, adjusting for age and sex. Thus, some trend toward an affective memory bias in MDD was observed, however, effect sizes were small. Finally, across individuals with a broad continuum of depressive symptoms, ranging from very low to very high, VAMT Bias scores were negatively associated with depressive symptoms at borderline significance.

In Part 1, we evaluated the psychometric properties of VAMT-26. The normal distribution provided a reasonable approximation to the distribution of IMR Total and IMR Positive and Negative recall scores, while STM and LTM outcomes were negatively skewed, although no VAMT-26 outcomes demonstrated ceiling effects, according to the definition suggested by Uttl (2005). The latter findings were expected since ceiling effects are usually avoided with lists containing more than 21 words (Uttl, 2005). About 7–9% of the participants recalled all positive and negative words within STM and LTM, most likely reflecting that our healthy participants all presented with no previous and current psychiatric illness, no family history of mood disorders and with high IQ scores and educational length. Our healthy, well-educated sample may have produced higher recall scores than would be obtained with the general Danish population, where we believe that ceiling effects would occur even more rarely.

As hypothesized, learning effects were observed after each of the five IMR list presentations, as seen for other list learning tests, for example the EVLT, CVLT-II (Delis et al., 2000), and RAVLT (Geffen et al., 1994). Participants recalled fewer words in the STM trial and in the LTM trial compared to the IMR5 trial, suggesting that the interference list and the 30 min interval before LTM successfully interfered with recall performances, as expected. Surprisingly, we observed an increase in the recall of words between the STM and LTM trial, which we did not observe for VAMT-24 (Jensen et al., 2015), despite the fact that VAMT-26 contains more words than VAMT-24. This increase involved a significantly larger recall of positive words within the LTM recall trial compared to the STM trial. The lack of an observable recall decline from STM Positive to LTM Positive could be related to the 30 min interval between STM and LTM recall, which may be too short a delay when examining healthy and well-educated individuals. Alternatively, or simultaneously, the neuropsychological tests employed during the 30 min delay as part of the standardized test sessions may not have interrupted LTM Positive recall sufficiently.

Primacy and recency effects, for the first and last three words, respectively, were reaffirmed for the A-26 list, supporting that recall is better for words at the beginning and end of a list compared to middle section words. To our knowledge, there is no consensus definition on the number of items to be used to assess primacy and recency effects. Although, our primacy and recency effects were similar to that reported for EVLT (Strauss and Allen, 2013), we cannot make a direct comparison as it was unclear how primacy and recency sections were defined.

Similar to the majority of memory tests (Delis et al., 2000; Fichman et al., 2010; Strauss and Allen, 2013; Jensen et al., 2015), VAMT-26 performance was influenced by demographic variables. We found that VAMT-26 performance across all VAMT-26 outcomes declined with age, in line with studies on verbal memory (Janowsky et al., 1996; Bopp and Verhaeghen, 2005; Kumar and Priyadarshi, 2013). The age effects observed in our study were present despite a relatively limited age range in our sample (age range: 18–54) and despite the fact that the participants had high IQ and educational scores, which has been shown to counteract age-related decline in verbal memory (Elwood, 1995; Clark et al., 2004). Women recalled more (here positive) words within LTM than men. These sex effects on verbal memory are consistent with results from previous verbal memory tests (Bleecker et al., 1988; Kramer et al., 1997; Delis et al., 2000; Strauss and Allen, 2013; Sundermann et al., 2016; Loprinzi and Frith, 2018) and add to a growing body of studies on epigenetic and neurodevelopmental research demonstrating that women may be more likely to develop superior verbal memory skills (Chung and Auger, 2013; Loprinzi and Frith, 2018). While higher IQ scores were associated with performance on VAMT-26 outcomes, educational scores were not, though our sample showed limited educational variation, and high IQ might decrease any effects of education on verbal recall (Strauss et al., 2006).

Our convergent validity tests consistently supported, to some degree, the validity of VAMT-26 scores, since these were positively associated with established scores on a neuropsychological instrument assessing non-affective working memory; LNS).

Total VAMT-26 scores showed acceptable 1-month test–retest precision. These results corroborate other verbal memory tests, e.g., the (C-AVLT) (Considine et al., 2017), the CVLT-II (Woods et al., 2006) and the RAVLT (Geffen et al., 1994). However, VAMT-26 Total scores demonstrated a pattern of learning effects, with improved performance on the test 1 month after first administration. At the retest session, individuals remembered on average 16.5 more words within IMR, 3.9 more words within STM, and 3.1 more words within LTM, compared to the baseline VAMT-26 test. The 90% CI for the bias did not contain 0, indicating learning effects from baseline to retest on the Total recall scores. Although the wide LOA intervals indicate a large individual variability in learning effects, i.e., a few individuals have no learning effects, while many individuals have large learning effects (e.g., some individuals have a learning effect that is double the average bias), all LOA intervals contained 0, indicating that there is no evidence that recall performance for all participants was significantly better at the second test. Taken together, while results on the test–retest precision analyses were satisfactory, results from limit of agreement analyses indicated a significant increase in recall at the second test session, supporting a learning effect. It is possible that a retest period of more than 1-month for testing with VAMT-26 will lower the learning effects, and we encourage future studies to assess the temporal stability of VAMT-26 Total scores at different time intervals.

In Part 2, we showed that data from VAMT-24 and VAMT-26 can be analyzed in a joint model by simply calculating the recall percentages within each outcome. In Part 3, we showed some trend toward a negative affective bias in verbal memory performance in MDD, however, the effect sizes were small. However, it is important to stress that the effect size of the affective memory bias found here in MDD was small, suggesting that the magnitude of this bias may be modest. For example, patients remembered on average 1.25 more negative words relative to positive words among the 50 words presented in IMR, and 0.5 more negative words relative to positive words among the 10 words presented within LTM compared to healthy controls. Whether the negative affective biases are clinically relevant, cannot be addressed with our data, but we do not think this is very likely, as the difference in recall of negative words relative to positive words in patients with MDD compared to healthy controls is very small, especially when considering the variability of VAMT Bias scores. In continuation of this, it is also possible that our large sample of patients with MDD and healthy controls has transformed small differences in affective memory bias into borderline significant differences. The affective memory biases in explicit non-self-referential in MDD converge with some previous findings in the depression literature (Watkins et al., 1992; Bradley et al., 1995; Neshat-Doost et al., 1998), and suggest that clinically depressed individuals preferentially recall negative information over positive information, while the healthy controls preferentially recall positive information (Ellwart et al., 2003). However, our findings contrast with other studies showing positive memory biases in patients with MDD (Danion et al., 1995; Calev, 1996; Zupan et al., 2017). Factors that could contribute to these discrepancies in findings on explicit affective memory biases in patients with MDD are small study samples, the use of different verbal memory tests, and different criteria for depression diagnosis. Across the entire sample (i.e., patients with MDD and healthy controls), we showed a statistical trend that VAMT Bias scores are negatively associated with depressive symptoms, suggesting that those with high negative memory bias are at higher risk of exhibiting depressive symptoms.

Future studies could consider using cognitive tasks assessing autobiographical and implicit memory as they may be more sensitive for measuring affective memory disturbances in MDD. Additionally, in this study we used a cross-sectional case-control design to examine affective bias in verbal memory in MDD-diagnosed patients compared to healthy controls. It is possible that VAMT-26 is more sensitive to detect mood-congruency effects in MDD in a within subject design (Jensen et al., 2015), or for example, before and after treatment with pharmacological treatments. This corroborates with our previous findings on VAMT-24, where we showed seasonal changes in negative affective bias in verbal memory performance in individuals with Seasonal Affective Disorder compared to healthy controls, using a longitudinal design (Jensen et al., 2015). Finally, future studies should examine whether mood-congruent bias in explicit non-referential affective memory can differentiate patients with MDD from healthy individuals, or instead characterize a subset of patients with MDD that respond differently to psychotherapy or pharmacological treatments, for example. The latter could reconcile with our findings of negative bias in verbal memory at borderline significance in patients with MDD.

### Methodological Considerations

We recommend and invite other researchers and clinicians to participate in further testing of the Danish VAMT versions and to the development of an English VAMT and versions in other languages. This study and the VAMT-26 test have several strengths. First, the relatively large sample size strengthens the statistical power to detect the impact of several covariates on verbal recall. In addition, the VAMT-26 list consists of both positive and negative words, which allows for an examination of preferential encoding and recall of certain types affective information. Finally, VAMT-26 words are equated on important stimuli features known to have an enhancing effect on memory, i.e., frequency of use in the Danish language, number of syllabics and that all words are nouns, common and non-taboo.

However, there are several limitations of VAMT-26. Firstly, larger and more representative samples, e.g., samples with larger educational and IQ variation, are needed to better estimate the influence of such variables on VAMT-26 outcomes. Secondly, the affective ratings of VAMT-26 words were established only on valence. Future studies should evaluate semantic relatedness and arousal, as such factors could affect the influence of affectivity on recall (Talmi and Moscovitch, 2004; Lewis et al., 2007; Mather and Sutherland, 2011; Bennion et al., 2013; Choi et al., 2013). Thirdly, the time interval between the first and second test to evaluate learning effects (∼1 month) was shorter than what might be ideal. Future test–retest studies with larger samples should be conducted to test learning effects of VAMT-26 scores over a longer time interval. Fourthly, in this paper, we propose a conversion algorithm to render VAMT scores comparable across different versions of VAMT. However, to directly compare the performance across VAMT test versions, future studies should employ both versions in the same sample of individuals. Fifthly, our large sample of patients and healthy controls may have amplified the detection of differences, emphasizing statistical differences that are not clinically relevant.

## Conclusion

In conclusion, we found that VAMT-26 demonstrated learning effects after each IMR list display, decreased recall for STM compared to IMR, as well as primacy and recency effects across IMR, STM and LTM trials. Positive and negative recall scores were internally consistent, and no test inherent affective biases were observed. VAMT-26 showed no ceiling effects. Variables, including age, sex and IQ scores were related to VAMT-26 recall performance, whereas educational level was not. VAMT-26 scores converged satisfactorily with a neuropsychological test associated with non-affective verbal memory. While retest precision was satisfactory over an approximately 1-month retest period, learning effects were not satisfactory but could likely be reduced with a longer test–retest interval. Data from VAMT-24 and VAMT-26 can be analyzed in a joint model by considering recall percentages instead of raw scores. Finally, patients diagnosed with MDD tended to remember more negative words relative to positive words compared to healthy controls at borderline significance. Thus, some trend toward mood-congruent bias in verbal memory in MDD was observed, however, effect sizes were small. We recommend VAMT-26 to be used in Danish research to study verbal affective recall, and in international studies after proper translation collaborations.

## Data Availability Statement

The datasets generated for this study are available on request to the corresponding author.

## Ethics Statement

The studies involving human participants were reviewed and approved by the Ethics Committee of Copenhagen and Frederiksberg, Denmark [protocol numbers: H-15013578 (*n* = 97), H-3-2013-100 (*n* = 39), H-2-2014-070 (*n* = 12), H-15001910 (*n* = 8), H-16026898 (*n* = 17), H-15017713 (*n* = 8) and H-1-2014-002 (*n* = 1)]. The patients/participants provided their written informed consent to participate in this study.

## Author Contributions

LH, VD, KK-F, DS, and GK designed the study. LH, SA, VD, and KK-F contributed to the data acquisition. LH, BO, CJ, VD, DS, and GK analyzed the data. LH, BO, CJ, SA, VD, KK-F, DS, and GK contributed to the interpretation of data. LH, BO, CJ, SA, VD, KK-F, DS, and GK wrote the original draft of manuscript. All authors approved the final version of the manuscript.

## Conflict of Interest

The authors declare that the research was conducted in the absence of any commercial or financial relationships that could be construed as a potential conflict of interest.

## Footnotes

1 http://ordnet.dk/korpusdk

## Supplementary Material

FIGURE S1 Q–Q plots of the residuals of the twelve univariate regression models used to model the mean and its variance of the nine VAMT-24 recall outcomes and their sum. The shaded area in dark gray 95% represents pointwise confidence intervals for the points of the QQ-curve (Oldford, 2016).

FIGURE S2 Distribution of VAMT-24 and VAMT-26 percentage recall rates. Histograms of the distribution of VAMT-24 and VAMT-26 recall-% scores adjusted for age, sex, IQ and educational level. The *y*-axis (density) indicates to the relative frequency normalized such that the area covered by the bars of the histogram equals 1.

FIGURE S3 Bland-Altman plots of test–retest VAMT-26 total scores. The graphs show on the *x*-axis the average value of the two test sessions and on the *y*-axis the difference between the two test sessions for IMR Total (left panel), STM Total (middle panel) and LTM Total (right panel). The red line indicates the expected difference between the two sessions, the blue and green dashed lines represent the lower and upper bound of the limits of agreement (i.e., 95% prediction interval for the difference between the test and retest values). The shaded areas represent the confidence intervals for the bias and bounds of the limits of agreement.

TABLE S1 Descriptive data for IMR 1–5 recall trials, primacy and recency effects.

TABLE S2 Established covariates of VAMT-26.
