# Supplementary figures and images for: Psychometric Properties of the Verbal Affective Memory Test-26 and Evaluation of Affective Biases in Major Depressive Disorder
Source: Front Psychol. 2020 Jun 5;11:961. doi: 10.3389/fpsyg.2020.00961 (PMC7289973; doi:10.3389/fpsyg.2020.00961)

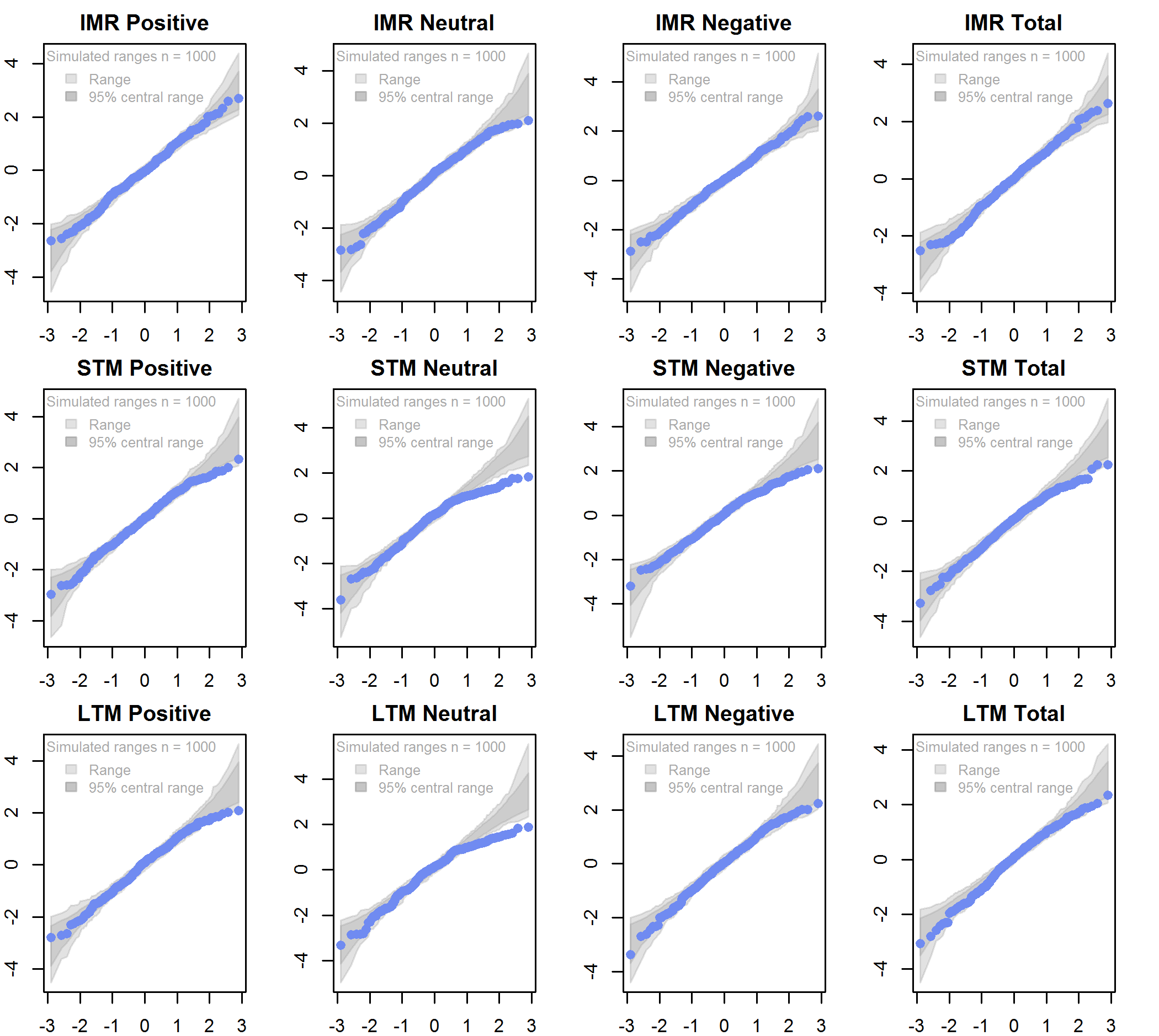

Supplement: FIGURE S1 — Q–Q plots of the residuals of the twelve univariate regression models used to model the mean and its variance of the nine VAMT-24 recall outcomes and their sum. The shaded area in dark gray 95% represents pointwise confidence intervals for the points of the QQ-curve (Oldford, 2016). [file Image_1.TIFF]

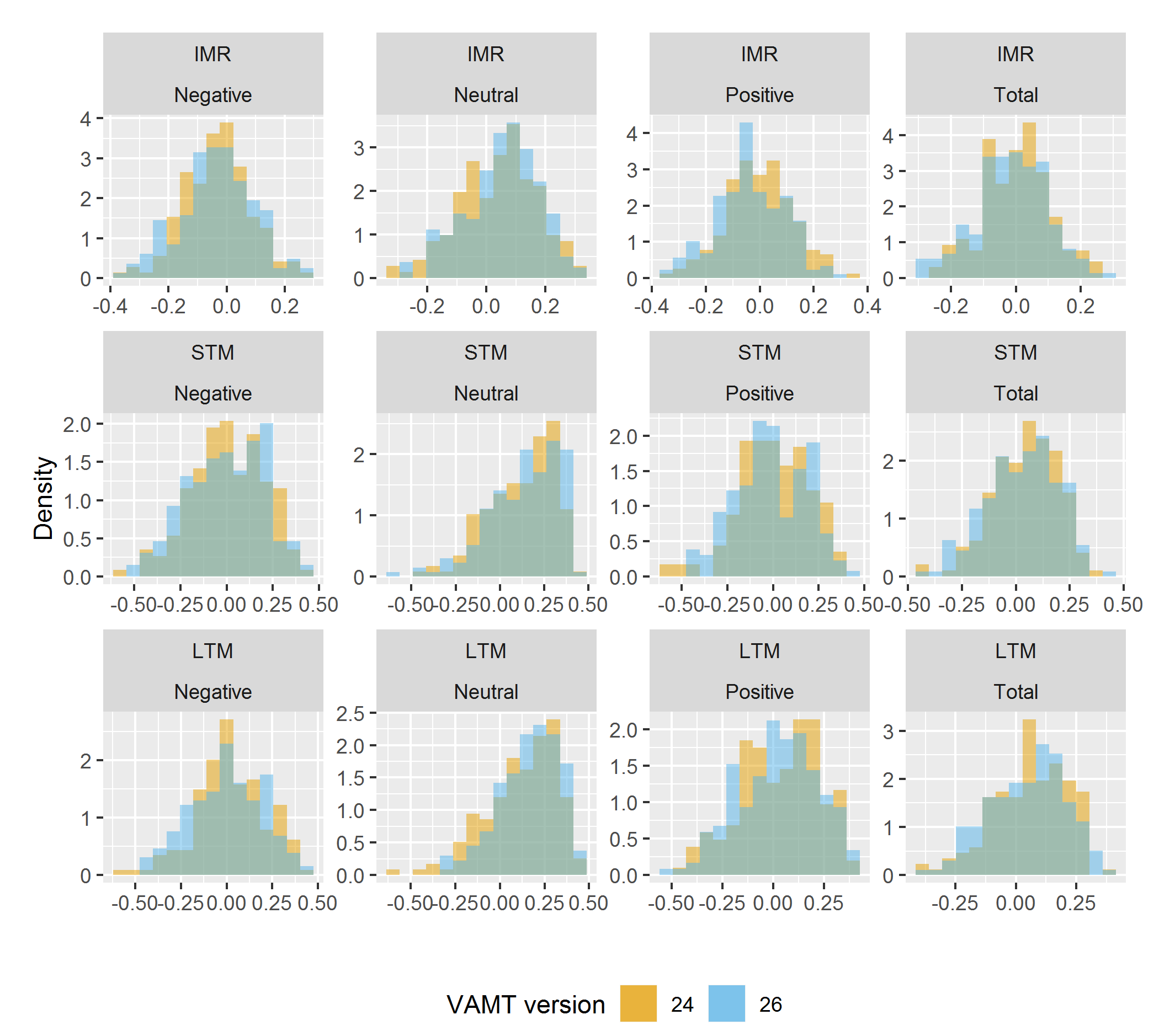

Supplement: FIGURE S2 — Distribution of VAMT-24 and VAMT-26 percentage recall rates. Histograms of the distribution of VAMT-24 and VAMT-26 recall-% scores adjusted for age, sex, IQ and educational level. The y-axis (density) indicates to the relative frequency normalized such that the area covered by the bars of the histogram equals 1. [file Image_2.TIFF]

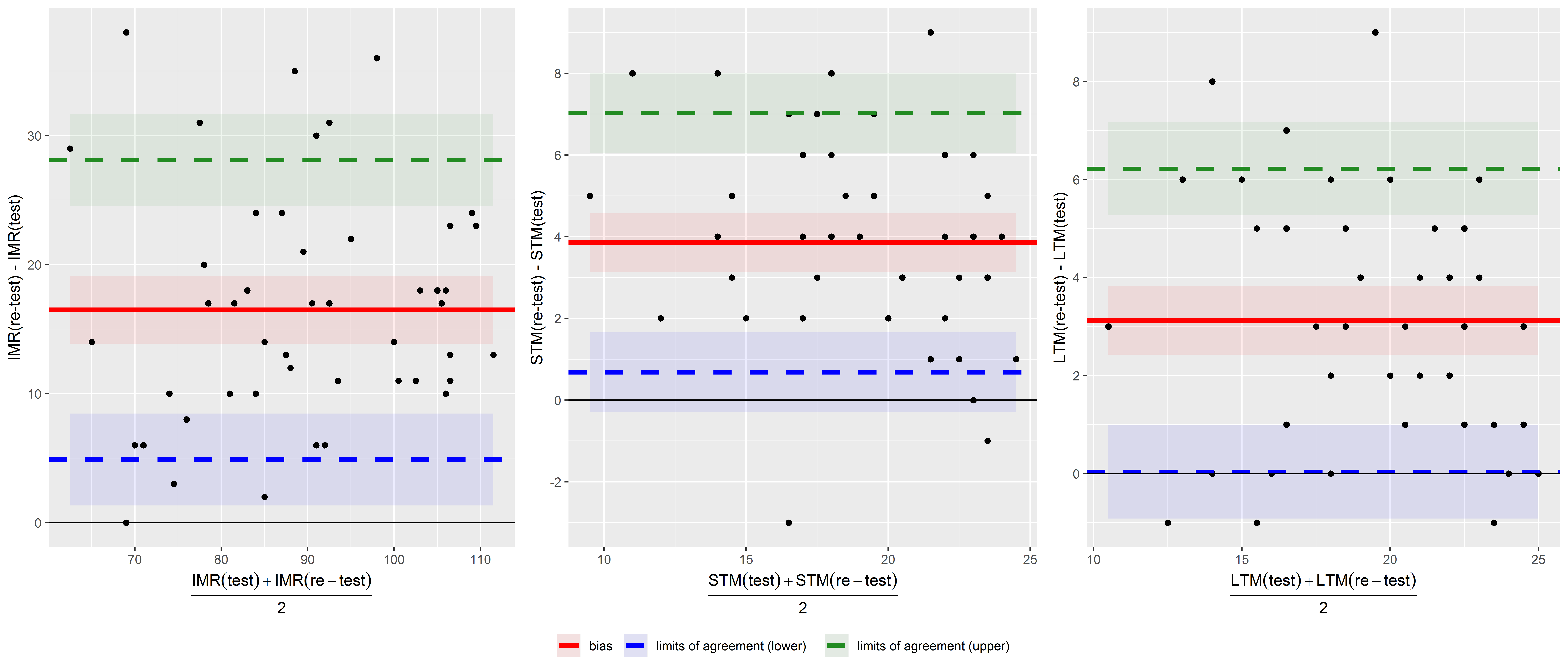

Supplement: FIGURE S3 — Bland-Altman plots of test–retest VAMT-26 total scores. The graphs show on the x-axis the average value of the two test sessions and on the y-axis the difference between the two test sessions for IMR Total (left panel), STM Total (middle panel) and LTM Total (right panel). The red line indicates the expected difference between the two sessions, the blue and green dashed lines represent the lower and upper bound of the limits of agreement (i.e., 95% prediction interval for the difference between the test and retest values). The shaded areas represent the confidence intervals for the bias and bounds of the limits of agreement. [file Image_3.TIFF]
