# Supplementary material for: Psychometric Properties of the Verbal Affective Memory Test-26 and Evaluation of Affective Biases in Major Depressive Disorder
Source: Front Psychol. 2020 Jun 5;11:961. doi: 10.3389/fpsyg.2020.00961 (PMC7289973; doi:10.3389/fpsyg.2020.00961)
Supplement: TABLE S1 — Descriptive data for IMR 1–5 recall trials, primacy and recency effects. [file Table_1.DOCX]

# Supplementary tables

| **Supplementary Table S1. Descriptive data for IMR 1–5 recall trials, primacy and recency effects** | | | | |
| --- | --- | --- | --- | --- |
| **Outcomes** | **Median** | **IQR** | **Range** | **Skewness** |
| Total IMR 1 | 10 | 4 | 3–18 | 0.21 |
| Total IMR 2 | 15 | 5 | 3–23 | -0.13 |
| Total IMR 3 | 17 | 5 | 2–26 | -0.56 |
| Total IMR 4 | 19 | 6 | 5–26 | -0.70 |
| Total IMR 5 | 20 | 6 | 4–26 | -0.79 |
|  |  |  |  |  |
| % recalled words in Primacy section (word 1-3) | 86.7 | 20 | 26.7-100 |  |
| % recalled words in Middle section (word 4-23) | 58.5 | 19.3 | 16-88 |  |
| % recalled words in Recency section (word 24-26) | 66.7 | 26.7 | 13.3-100 |  |
| **Note:** Median, interquartile range, minimum and maximum scores and skewness scores for IMR trials 1–5, STM trial and LTM trial as well as for % recalled words in the Primacy section, Middle section and Recency section. IMR=Immediate recall, STM=Short-term memory, LTM=Long-term memory, IQR=Interquartile range. | | | | |

| **Supplementary Table S2. Established covariates of VAMT-26** | | | | | |  |  |  |  |  |  |
| --- | --- | --- | --- | --- | --- | --- | --- | --- | --- | --- | --- |
|  | **Sex** | |  | **Age** | |  | **Education** | |  | **IQ** | |
|  | *Beta*  *coefficients* | *Adjusted p-values* |  | *Beta*  *coefficients* | *Adjusted p-values* |  | *Beta*  *coefficients* | *Adjusted p-values* |  | *Beta*  *coefficients* | *Adjusted p-values* |
| IMR Total | -5.3 | 0.15 |  | -0.6 | <0.0001 |  | 0.9 | 0.97 |  | 0.6 | <0.001 |
| STM Total | -1.0 | 0.30 |  | -0.2 | <0.0001 |  | 0.5 | 0.34 |  | 0.1 | 0.01 |
| LTM Total | -1.9 | 0.03 |  | -0.2 | <0.001 |  | 0.4 | 0.52 |  | 0.1 | 0.01 |
| IMR Positive | -2.2 | 0.22 |  | -0.2 | 0.003 |  | 0.1 | 0.76 |  | 0.1 | <0,01 |
| IMR Negative | -1.5 | 0.37 |  | -0.3 | <0.0001 |  | 0.3 | 0.73 |  | 0.2 | <0.01 |
| STM Positive | -0.6 | 0.27 |  | -0.1 | <0.0001 |  | 0.9 | 0.62 |  | 0.1 | 0.01 |
| STM Negative | -0.1 | 0.67 |  | -0.1 | <0.001 |  | 0.2 | 0.54 |  | 0.05 | 0.08 |
| LTM Positive | -1.0 | 0.01 |  | -0.1 | <0.001 |  | 0.2 | 0.55 |  | 0.05 | 0.02 |
| LTM Negative | -0.5 | 0.34 |  | -0.1 | 0.006 |  | 0.2 | 0.63 |  | 0.1 | 0.04 |
| **Note.** The estimated associations between primary VAMT-26 outcomes and established covariates, i.e. sex, age, education and IQ. Beta coefficients denotes the estimate of the linear regression model for a given descriptive variable. Within each set of regression analyses on sex, age, education and IQ, *p*-values are adjusted for nine tests, respectively, using the Bonferroni-Holm adjustment procedure. IQ score is assessed with the Reynolds Intellectual Screening Test and education score is measured with the Online Stimulant and Family History Assessment Module. IMR=Immediate recall, STM=Short-term memory, LTM=Long-term memory, IQ=Intelligence Quotient. | | | | | | | | | | | |
|  |  |  |  |  |  |  |  |  |  |  |  |
